# Supplementary material for: Abnormal Cellular Phenotypes Induced by Three TMPO/LAP2 Variants Identified in Men with Cardiomyopathies
Source: Cells. 2023 Jan 16;12(2):337. doi: 10.3390/cells12020337 (PMC9857342; doi:10.3390/cells12020337)
Supplement: Supplementary file 1 [file cells-12-00337-s001.zip › cells-2131610-supplementary/S1_File_Vadrot et al_Cells.pdf]

File S1

1- Cardiomyopathy Targeted Panel Sequencing

1a - List of genes sequenced in their coding regions.

Genes	Reference seq	Genes	Reference seq
ABBC9	NM_020297	MYL2	NM_000432
ACTC1	NM_005159	MYL3	NM_000258
ACTN2	NM_001103	MYLK2	NM_033118
ANKRD1	NM_014391	MYOZ2	NM_016599
BAG3	NM_004281	MYPN	NM_032578
CRYAB	NM_001885	NEBL	NM_006393
CSRP3	NM_003476	NEXN	NM_144573
DES	NM_001927	NKX2-5	NM_004387
DSG2	NM_001943	PLN	NM_002667
DSP	NM_004415	PRKAG2	NM_016203
DTNA	NM_001390	RBM20	NM_001134363
EMD	NM_000117	RYR2	NM_001035
FHL1	NM_001159702	SCN5A	NM_198056
FLNC	NM_001458	TAZ	NM_000116
GATA4	NM_002052	TCAP	NM_003673
GLA	NM_000169	TMPO	NM_003276
LAMA4	NM_001105206	TNNC1	NM_003280
LAMP2	NM_002294	TNNI3	NM_000363
LDB3	NM_007078	TNNT2	NM_001001430
LMNA	NM_170707	TPM1	NM_001018005
MYBPC3	NM_000256	TTN	NM_001256850
MYH6	NM_002471	TTR	NM_000371
MYH7	NM_000257	VCL	NM_014000

1b - Method for gene sequencing.

Custom targeted genes enrichment and DNA library preparation were performed using the NimbleGen EZ choice probes® and Kappa Library preparation kit according to the manufacturer's instructions (NimbleGen®, Roche Diagnostics, Madison, USA). The targeted regions (coding regions and flanking intronic regions (± 20 bp) were sequenced using the Illumina MiSeq platform on a 500 cycle Flow Cell (Illumina, Santa Cruz, USA) and MiSeq Software generated FASTQ format files after demultiplexing patients' sequences. Merged single reads and paired-end reads were then aligned on Hg19 human reference genome, using BWA-MEM. Variant calling was performed using the GATK Haplotype Caller program then annotated using ANNOVAR. Written informed consent was obtained from all study participants before inclusion in the study.
