# Supplementary material for: Abnormal Cellular Phenotypes Induced by Three TMPO/LAP2 Variants Identified in Men with Cardiomyopathies
Source: Cells. 2023 Jan 16;12(2):337. doi: 10.3390/cells12020337 (PMC9857342; doi:10.3390/cells12020337)
Supplement: Supplementary file 1 [file cells-12-00337-s001.zip › cells-2131610-supplementary/S2_File_Vadrot et al_Cells.pdf]

File S2 List of Primers

Primers used for generation of plasmids encoding LAP2 missense variants

p.(Leu124Phe) forward 5’ gaagatcttttggatcagtttgtgaaatacggagtg 3’
reverse 5’ cactccgtatttcacaaactgatccaaaagatcttc 3’;
p.(Ala240Thr) forward 5’ gcagtaccgaactacagacagctaagaaagtacatac 3’
reverse 5’ gtatgtactttcttagctgtctgtagttcggtactgc 3’
p.(Arg690Cys) forward 5’ ggaggagaagtatgcaaagtaattaaaaagtgtggaaataaacac 3’
reverse 5’ gtgtttatttccacactttttaattactttgcatacttctcctcc 3’;

Primers used for generation of plasmids encoding the LAP2α variant p.(Gly395Glufs*11)

p.(Gly395Glufs*11) forward 5’ ggtaggtagtttgcctgaacttctaactctatgc 3’
reverse 5’ gcatagagttagaagttcaggcaaactacctacc 3’.

p.(Gly395Glufs11)-DDK forward 5’ – gccgcc<u>gcgatcgc</u>catgccggagttcctggaag – 3’
reverse 5’- ctgctcga<u>gcggccgc</u>gtcatccagtgggggcatag -3’

Primers used to amplify by PCR the cDNA encoding BAF

for further ligation into the EcoR1 and XBa1 sites of pcDNA-3HA (Addgene):

BAF forward : 5’ -gcgaattcacaacctcccaaaagcaccg-3’
reverse : 5’ -cgtctagactacaagaaggcatcacacc-3’

Primers used for qRT-PCR

<u>Human primer sequences</u> :

HPRT forward : 5’ –accccacgaagtgttggata-3’
reverse : 5’ –aagcagatggccacagaact-3’
GAPDH forward : 5’ –gtgaaggtcggagtcaacgg-3’
reverse : 5’ –acatgtaaaccatgtagttgaggtc-3’

18S rRNA	forward : 5'-acggaccagagcgaaagcat-3'
	reverse : 5'- ggacatctaagggcatcacagac-3'
Beta Actin	forward : 5'-atcatgtttgagaccttcaa-3'
	reverse : 5'-ttgcgctcaggaggagcaat-3'
Rpl19	forward : 5'-tcaaaaacaagcggattctca-3'
	reverse : 5'- gcgtgcttccttggtcttag-3'
TBP	forward : 5'- gggttttccagctaagttcttg-3'
	reverse : 5'-agcacaaggccttctaacctt-3'
PPIA	forward : 5'-ctggttccttctgcgtgaat-3'
	reverse : 5'- aaccagacaacacacacaagactga-3'
LAP2α (1)	forward 5'-gtgacaatgaagaaggaaag-3'
	reverse 5'-ggtaggtctcccttagaagt-3'
LAP2α (2)	forward 5'-gaagaaggaaagaagaaagaacac-3'
	Reverse 5'-ttagctgcctgtagttcggt-3'

Rat primer sequences (H9C2 cells)

GAPDH	forward : 5'-aggagtaagaaaccctggac-3'
	reverse : 5'-ctgggatggaattgtgag-3'
HPRT	forward : 5'-cccagcgtcgtgattagtgatg-3'
	reverse : 5'-ttcagtcctgtccataatcagtcc-3'
TBP	forward : 5'-caccgtgaatcttggctgtaaac -3'
	reverse : 5'-cgcagttgttcgtggctctc -3'
HMBS	forward : 5'-tctagatggctcagatagcatgca-3'
	reverse : 5'-tggaccatcttcttgctgaaca-3'
Actin B	forward : 5'- aagtccctcaccctcccaaaag -3'
	reverse : 5'- aagcaatgctgtcaccttccc -3'
YWHAZ	forward : 5'- gatgaagccattgctgaacttg -3'
	reverse : 5'- gtctccttgggtatccgatgtc -3'
