# Supplementary material for: Abnormal Cellular Phenotypes Induced by Three TMPO/LAP2 Variants Identified in Men with Cardiomyopathies
Source: Cells. 2023 Jan 16;12(2):337. doi: 10.3390/cells12020337 (PMC9857342; doi:10.3390/cells12020337)
Supplement: Supplementary file 1 [file cells-12-00337-s001.zip › cells-2131610-supplementary/S3_File_Vadrot et al_Cells.pdf]

File S3

List of Antibodies Used for Immunofluorescence and Western Blot Analysis

Primary antibodies used for Immunofluorescence (IF) analysis were: rabbit anti BAF (dilution 1:40; sc-33787, SantaCruz Biotechnolgy, Inc), rabbit anti Calnexin (dilution 1:200; ab22595, Abcam, UK), rabbit anti FLAG (DDK) (dilution 1:300; F7425, Sigma-Aldrich, USA), rabbit anti HMGN5 (NSBP1) (dilution 1:200; orb11169, Biorbyt LLC, USA), rabbit anti-lamin A/C (dilution 1:400) [1], rabbit anti lamin B1(dilution 1:300) [1], rabbit anti-LAP2α (dilution 1:300; IQ175, Immuquest, UK), rabbit anti LAP2β (dilution 1:250; PA5-52519, ThermoFisher Scientific, USA), mouse anti DDK (FLAG) (dilution 1:600; TA50011, OriGene, USA), mouse anti emerin (dilution 1:30; 4G5, Novocastra Laboratories Ltd, UK) and mouse anti HA (dilution 1:150; H9658, Sigma-Aldrich, USA). Actin fibers were stained with Phalloidin conjugated to FITC (dilution 1:100; P5282, Sigma-Aldrich, USA). Secondary antibodies were Affinipure donkey anti-rabbit coupled to Cy2 (711-225-152) or DyLight 594 (711-515-152), and Affinipure donkey anti-mouse coupled to Cy2 (715-225-150) or DyLight 594 (715-515-150) (Jackson ImmunoResearch, Europe), diluted 1:100 to 1:200.

Primary antibodies used for immunoblotting (Iblot) analysis were: rabbit anti-GAPDH (dilution 1:5000; G9545, Sigma-Aldrich, USA), rabbit anti HA (dilution 1:400; sc-805, SantaCruz Biotechnolgy, Inc), rabbit anti-LAP2α (dilution 1:2000; IQ175, Immuquest, UK), rabbit anti LAP2β (dilution 1:400; PA5-52519, ThermoFisher Scientific, USA), rabbit anti TMPO/LAP2 (Proteintech, Europe), rabbit anti Histone H4 (dilution 1:200; ab10158 Abcam, UK), mouse anti actin (dilution 1:5000; A3853, Sigma-Aldrich, USA), mouse anti DDK (dilution 1:2000; TA50011, OriGene, USA), mouse anti HA (dilution 1:500; H9658, Sigma-Aldrich, USA), mouse anti Tubulin (dilution 1:500; (4G1) sc-58666, SantaCruz Biotechnolgy, Inc). Secondary antibodies were goat anti-rabbit (W4011) and goat anti-mouse (W4021) coupled to horseradish peroxidase (Promega, France), diluted 1:1000 to 1:2000.

Reference

1. Favreau C, Higuet D, Courvalin J-C, Buendia B. Expression of a mutant lamin A that causes Emery-Dreifuss muscular dystrophy inhibits in vitro differentiation of C2C12 myoblasts. *Mol Cell Biol*. **2004**, *24*, 1481–1492.
