# Supplementary material for: Abnormal Cellular Phenotypes Induced by Three TMPO/LAP2 Variants Identified in Men with Cardiomyopathies
Source: Cells. 2023 Jan 16;12(2):337. doi: 10.3390/cells12020337 (PMC9857342; doi:10.3390/cells12020337)
Supplement: Supplementary file 1 [file cells-12-00337-s001.zip › cells-2131610-supplementary/Supplemental Figure S1,2-Legend.pdf]

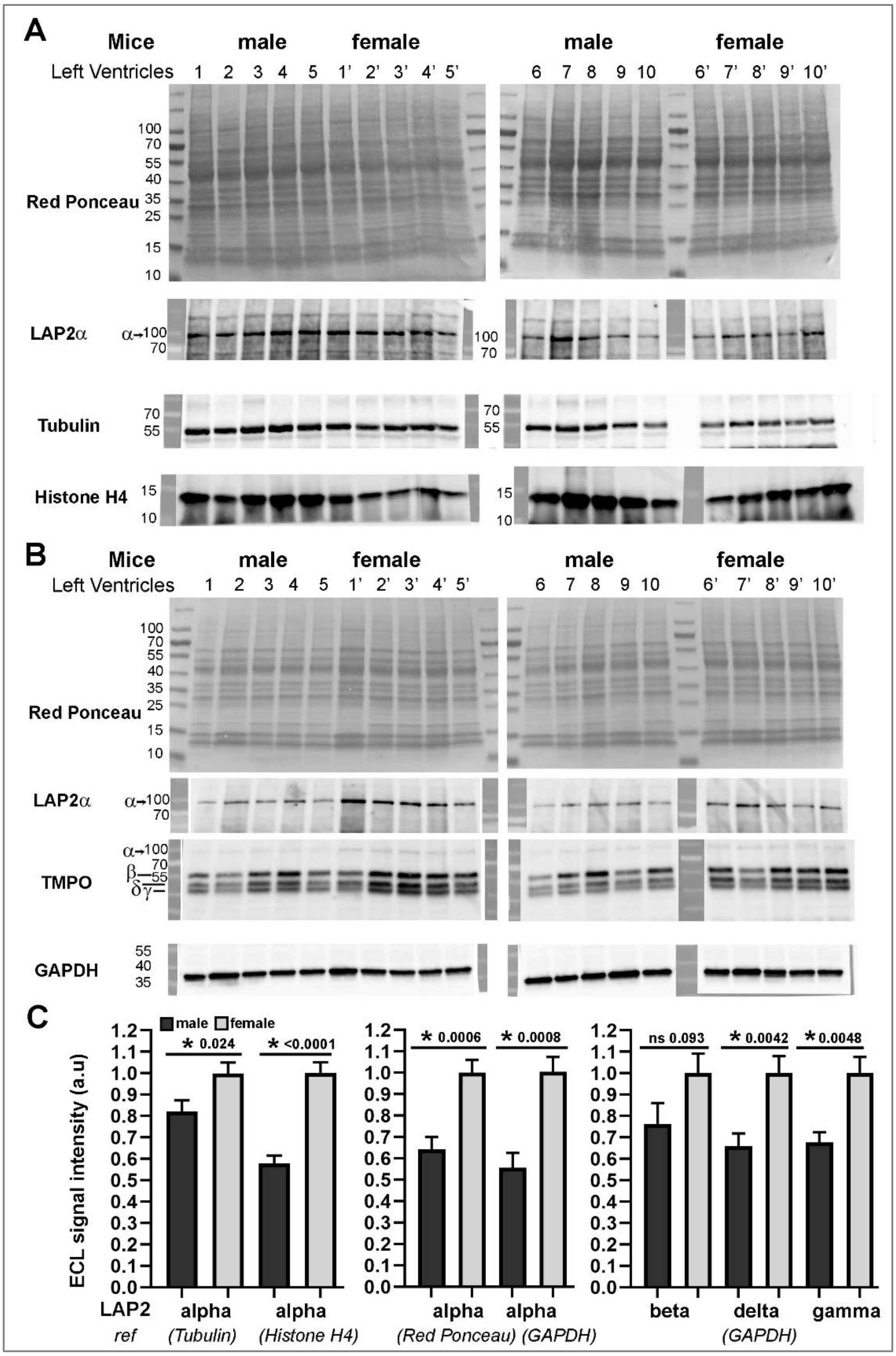

Figure S1. Relative expression level of LAP2 proteins in males vs females mice aged of 9 weeks. Western blot on whole extracts of LV from 10 male mice (1–10) and 10 female mice (1′–10′). (**A**), In a first set of gels, western blots were stained with red ponceau and incubated with anti-LAP2α antibodies, while Tubulin and Histone H4 were used to normalize ECL signals. (**B**), In a second set of gels, western blots were stained with red ponceau and incubated first with anti-LAP2α antibodies and subsequently with TMPO antibodies (that recognize all LAP2 proteins), while Red Ponceaui staining or GAPDH were used

to normalize ECL signals. (**C**) The graph shows ECL signal quantification related to (**A**) and (**B**) (mean ± s.e.m; n = 10). For the statistic analyses, precise p values are indicated.

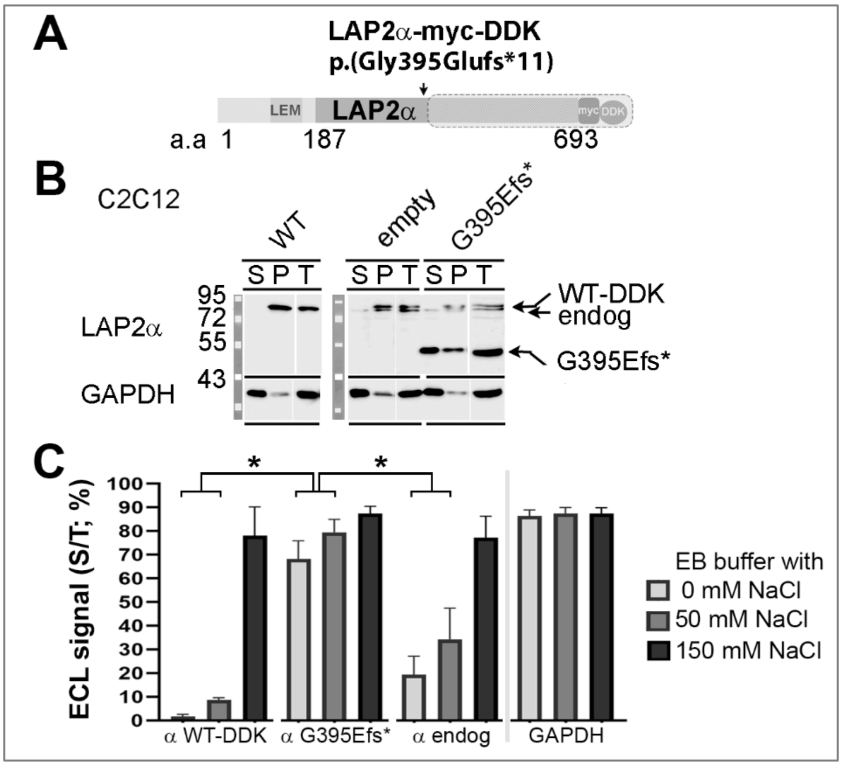

Figure S2. High solubility properties of truncated LAP2*α* in cells**.** (**A**) Schematic secondary structure of exogenous p.(Gly395Glufs*11) (G395Efs*) LAP2*α* proteins. (**B**) LAP2*α* western blot on whole cell extracts of C2C12 cells overexpressing either wild-type (WT-DDK) or mutant (G395Efs*) LAP2*α* or transfected with an empty vector (empty). LAP2*α* and GAPDH western blots on fractions of proteins released in the Supernatant (S) or remaining in the pellet (P) after extraction in the presence of Triton as well as total proteins (T) of C2C12 cells transfected with either an empty plasmid (empty) or plasmids encoding WT-DDK or G395Efs* LAP2*α* as indicated. (**C**) Relative quantitative amount of the pool of LAP2*α* proteins (*α* WT-DDK, *α* G395Efs*, *α* endogenous) released in the supernatant after extraction of C2C12 cells in a buffer containing Triton and either 0 mM, 50 mM or 100 mM NaCl concentrations. The graph shows ECL signal quantification (mean ± sem) of LAP2*α* proteins detected in the supernatant fraction as compared to total proteins (n = 3 experiments). GAPDH was used as a control cytoplasmic protein.
